# Sedentary time among spouses: a cross-sectional study exploring associations in sedentary time and behaviour in parents of 5 and 6 year old children

**DOI:** 10.1186/s13104-015-1758-8

**Published:** 2015-12-15

**Authors:** Lesley Wood, Russell Jago, Simon J. Sebire, Jesmond Zahra, Janice L. Thompson

**Affiliations:** Centre for Exercise, Nutrition and Health Sciences, School for Policy Studies, University of Bristol, 8 Priory Road, Bristol, BS8 1TZ UK; School of Sport, Exercise and Rehabilitation Sciences, University of Birmingham, Birmingham, UK

**Keywords:** Parent, Spousal behaviour, Cross-sectional, Sedentary time, Screen viewing

## Abstract

**Background:**

Sedentary time is associated with obesity and is a risk factor for other adverse health outcomes. We examined how sedentary time and screen viewing (SV) behaviours in parents of young children are associated and whether associations differed for weekdays versus weekend days.

**Methods:**

Data were from a cross sectional study (B-ProAct1v) based in Bristol, UK investigating associations between physical activity and SV in children and parents. Parents were eligible for analysis if they and their partner had both provided valid accelerometer data (290 dyads) or had both provided valid screen-viewing data (325 dyads). Multivariable regression models were used to examine associations of (a) sedentary behaviours and (b) self-reported time spent on weekdays and weekend days watching TV, using a PC, and using a phone in the dyads. Models were adjusted for the number of media items in the house, mothers’ age and body mass index, and household index of multiple deprivation.

**Results:**

Sedentary behaviour was lower at weekends than on weekdays for fathers and mothers. In contrast, the proportion of parents watching at least 2 h TV was higher on weekend days than on weekdays. Adjusted multivariable linear regression models suggested that 3 min of sedentary time on weekend days in fathers were associated with an additional minute of mothers’ sedentary time (B 0.38; 95 % CI 0.26 to 0.49). Logistic regression indicated that mothers’ screen use was positively predicted by fathers’ use (e.g., the odds of a mother watching more than 2 h TV on a weekend day were increased fivefold if the father also watched this amount OR 5.09, 95 % CI 3.30 to 7.86), except for PC use at weekends where the association was reversed and the odds of mothers using a PC for more than 30 min per weekend day was halved if the father used a PC for this amount of time (OR 0.45, 95 % CI 0.22 to 0.94).

**Conclusions:**

Programmes that encourage at least one adult in the household to decrease sedentary behaviour and become more active, particularly at weekends, should be developed.

## Background

Sedentary behaviour is characterised by an energy expenditure of below 1.5 metabolic equivalents whilst sitting or lying [1]. Sedentary behaviour is associated with obesity [2] and other adverse health outcomes. Common sedentary behaviours include screen viewing (SV) such as watching television, using computers, tablets and smartphones, and playing video games. Greater time spent in this type of SV behaviour has been associated with an increased risk for cardiovascular disease, type-2 diabetes and all-cause mortality among adults [3].

Body mass index (BMI) in co-habiting couples is significantly correlated at 0.266 [4] and if one spouse becomes obese, the other spouse is 37 % more likely to become obese [5]. This association could be due to a number of factors, including one partner influencing eating and exercise behaviours in either direction. Although some studies have investigated associations of PA in young couples [6, 7] there is little evidence about either SV or sedentary time in couples who have young children.

Adults’ PA and SV patterns have been shown to differ across the week [8]. Whether this applies to sedentary time and whether this type of behaviour is associated in co-habiting parents of young children is unknown. Understanding how patterns of associations vary across the week is important for identifying an appropriate time to modify behaviours. Thus, the aim of the current study was to examine how SV and sedentary time of fathers is associated with that of the mother within the same household and also whether associations differed on weekdays versus weekend days.

## Methods

**Fig. 1:**
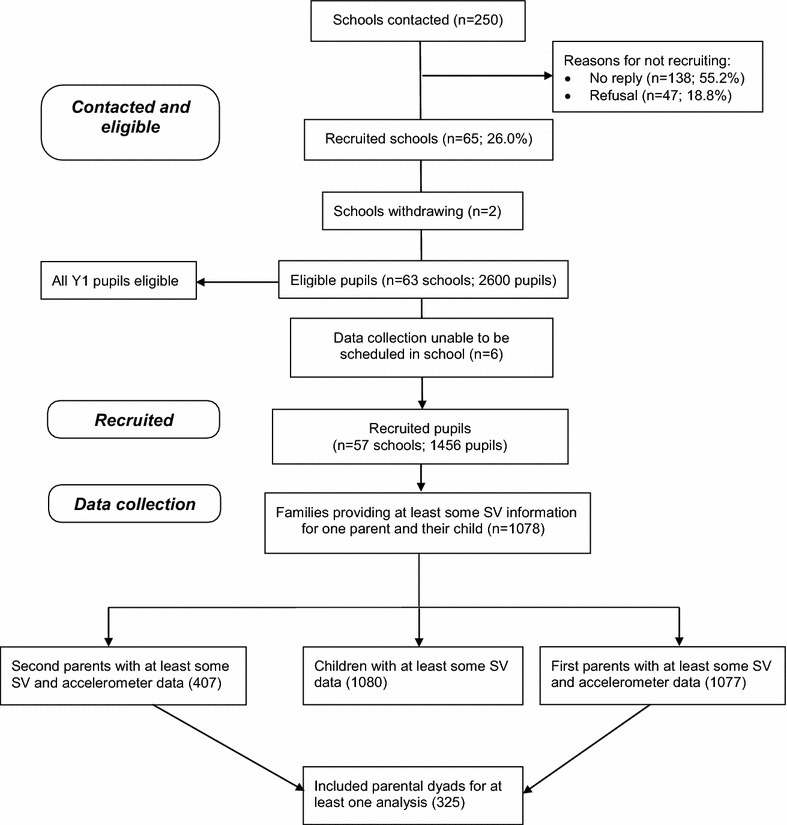
Study flow of participants

Data are from a cross-sectional study (B-ProAct1v) which aimed to identify factors associated with PA and SV among children in their second year of schooling. Full details of the study have been published elsewhere [9, 10]. Briefly, children aged five to six years and their parents were recruited from 57 primary schools within 20 miles of Bristol between Jan 2012 and May 2012. Of the recruited 1456 families, 1267 pupils and at least one parent returned an accelerometer. Consistent with the STROBE guidelines, Fig. 1 provides a detailed presentation of the number of contacted and eligible participants, reasons for school non-participation, recruitment levels and the size of the sample which met our analysis inclusion criteria [11]. The study was approved by the School for Policy Studies ethics committee at the University of Bristol and written informed consent was obtained for all participants.

To be included in analysis for this element of the project, both parents were required to wear an Actigraph GT3X accelerometer for five days, including two weekend days, and were also asked to provide information about their SV time on weekdays and weekend days. Separate questions were asked for the following SV items: TV, computer/laptop, games console, tablet and smartphone (except for time spent texting or talking). For each item, the parent was asked to report the time s/he spent using it for (a) a normal weekday and (b) a normal weekend day, with response options: none; 1–30 min; 31–1 h; 1–2 h; 2–3 h; 3–4 h; 4 h or more. The number of media items in the household was also reported.

Home postcode was reported and an index of multiple deprivation (IMD) score, using the English Indices of Deprivation (http://data.gov.uk/dataset/index-of-multiple-deprivation), was derived. A higher IMD score indicates a greater level of deprivation.

### Data preparation

For this current study, parents were considered to have met the criteria for accelerometer data to be valid if they provided at least three days of valid data, where a valid day was defined as the provision of at least 500 min of data. Periods of ≥60 min of zero values, with an allowance of up to 2 min of interruptions, were defined as accelerometer “non-wear” time and were removed from the analyses [12]. Sedentary time was determined from accelerometer data using a threshold of <100 counts per minute [13].

TV viewing behaviour on week and weekend days was dichotomized into <2 h per day and ≥2 h per day. Computer use on weekdays and weekend days was divided into two categories (≤30 min per day; >30 min per day). Smartphone use was dichotomized into ‘no use’ and ‘some use’.

To be included in the analysis for this paper, a male and a female parent were required to have provided accelerometer data for at least 2 valid weekdays, and at least 1 valid weekend day [12]. In addition, both members of the dyad needed to have completed the screen viewing elements of the questionnaire. Mothers were also required to have provided complete information for potential explanatory variables (age, height and weight). Mothers’ body mass index (BMI = kg/m^2^) was calculated using their self-reported height and weight.

### Statistical analysis

Descriptive statistics (means and standard deviations, or percentages) were calculated. Differences in key variables between parents who did and did not provide sufficient information to be included in this analysis were examined using Student t tests.

Linear regression models were used to estimate the extent to which fathers’ sedentary time was associated with that of mothers on both weekdays and weekend days. Logistic regression models were used to investigate whether mother’s SV time for each screen type on (a) weekdays and (b) weekend days was associated with father’s time spent using the same SV device. All models were subsequently adjusted for the total number of media items reported to be in the house, mother’s age and BMI, and the household IMD. Mother’s BMI was included as there is evidence of an association between BMI and activity/sedentary behaviour [4]. All analyses were performed in Stata version 12.0 [14]. Confidence intervals (CI) for all models were based on robust standard errors to account of the clustered nature of recruitment at school level. Robust standard errors are used to allow for clustering and the fact that the parameter estimates are not based on a full probability model [15]. More specifically in this case, the method allows for the errors to vary between clusters (in this case between schools).

## Results

Overall, 290 dyads were included in the analyses examining associations between mothers’ and fathers’ weekday sedentary time and 301 dyads for weekend sedentary time. For associations between parents screen use time, 325 (324 for weekday PC use) were included (Fig. 1).

Mothers who were excluded from analyses because of incomplete data were younger and were from households with higher IMD scores than those who were included. They also spent less time in sedentary time on weekdays and weekend days than mothers who were included. Excluded fathers were similar to those who were included in terms of age, BMI, and weekday sedentary time, but spent less time engaged in sedentary time on weekend days (Table 1).

**Table 1:** Characteristics of participants and households that were included in the overall analysis compared with those who were excluded

	Included			Excluded			p*
	N	Mean	SD	N	Mean	SD	
Mothers’ age	325	38.1	5.3	531	36.8	5.6	0.002
Mothers’ BMI	325	25.1	4.5	540	25.0	4.5	0.598
Fathers’ age	309	40.0	5.7	236	39.2	6.2	0.117
Fathers’ BMI	311	26.2	3.7	237	26.5	4.1	0.423
Household IMD^a^	325	12.6	10.0	846	15.9	13.7	<0.001
Number of TVs in household	325	2.2	1.2	741	2.4	1.2	0.004
Number of DVDs in household	325	1.5	0.91	741	1.6	0.98	0.014
Number of Digital TV recorders	325	0.94	0.68	741	0.98	0.77	0.361
Number of music players	325	2.6	1.8	741	2.2	1.6	<0.001
Number of desktop PCs	325	0.60	0.65	741	0.51	0.65	0.045
Number of laptops	325	1.3	0.83	741	1.2	0.83	0.072
Number of tablets	325	0.55	0.81	741	0.48	0.69	0.141
Number of games consoles	325	1.1	0.91	741	1.2	0.89	0.184
Number of smartphones	325	1.5	1.0	741	1.4	1.0	0.169
Number of handheld consoles	325	1.1	1.1	741	1.2	1.1	0.555
Total number of TV items	325	4.6	2.2	741	5.0	2.5	0.007
Total number of computers	325	2.4	1.5	741	2.2	1.3	0.003
Total number of games consoles	325	2.2	1.8	741	2.3	1.7	0.193
Total number of SV devices	325	13.3	5.3	741	13.0	5.4	0.409
Mothers’ sedentary time (min) weekdays	290	545.0	86.6	576	511.7	90.3	<0.001
Fathers’ sedentary time (min) weekdays	290	572.3	104.7	223	566.6	98.9	0.528
Mothers’ sedentary time (min) weekend	301	507.9	94.7	530	469.1	95.6	<0.001
Fathers’ sedentary time (min) weekend	301	525.3	93.8	219	506.2	96.5	0.024

^*^p value from t test

^a^A higher value indicates greater deprivation

Households of included participants had an average of 2.2 televisions which increased to 4.6 when all TV associated devices (such as DVD players) were included. For included parents, sedentary time was lower at weekends than on weekdays for both mothers (507 vs 545 min) and fathers (525 vs 572 min) (Table 1).

Almost twice as many fathers spent at least 2 h watching TV on weekend days (59 %) compared with weekdays (30 %) (Table 2). This difference was smaller for mothers (51 vs 28 %). Although fewer mothers used a smart phone compared with fathers, use was similar on weekdays and weekend days, with just over half of mothers, and just over two-thirds of fathers reporting some use across both types of day.

**Table 2:** Screen viewing time, PC use and smart-phone use for mothers and fathers on weekdays and weekend days

	<2 h per day		≥2 h per day	
	n	%	n	%
Mothers’ weekday TV (n = 325)	233	71.7	92	28.3
Fathers’ weekday TV (n = 325)	229	70.5	96	29.5
Mothers’ weekend day TV (n = 325)	159	48.9	166	51.1
Fathers’ weekend day TV (n = 325)	135	41.5	190	58.5

	<30 min per day		≥30 min per day	
	n	%	n	%
Mothers’ weekday PC (n = 324)	130	40.1	194	59.9
Fathers’ weekday PC (n = 324)	119	36.7	205	63.3
Mothers’ weekend day PC (n = 325)	51	15.7	274	84.3
Fathers’ weekend day PC (n = 325)	106	32.6	219	67.4

	No use		Some use	
	n	%	n	%
Mothers’ weekday phone use (n = 325)	140	43.1	185	56.9
Fathers’ weekday phone use (n = 325)	98	30.2	227	69.8
Mothers’ weekend day phone use (n = 325)	146	44.9	179	55.1
Fathers’ weekend day phone use (n = 325)	94	28.9	231	71.1

Results from linear regression models suggest that, after controlling for mother’s age, BMI, household IMD and the number of SV devices in the house, each additional 5 min spent in sedentary time by the father on a weekday was associated with an additional minute of sedentary time by mothers (B 0.20; 95 % CI 0.09 to 0.32). At weekends, an increment of just under 3 min in fathers’ sedentary time was associated with a 1 min increment in mothers’ time (B 0.38; 95 % CI 0.26 to 0.49) (Table 3). The number of media devices in the household was not associated with this increment on either weekdays or weekend days.

**Table 3:** Linear regression of mothers’ sedentary time predicted by fathers’ sedentary time

	Unadjusted			Adjusted^a^		
	Coeff (B)	95 % CI	p	Coeff (B)	95 % CI	p
Mothers’ sedentary time weekdays (n = 290)						
Fathers’ sedentary time weekday (mins)	0.21	0.10 to 0.32	<0.001	0.20	0.09 to 0.32	0.001
N media items in household				0.60	−1.50 to 2.70	0.569
	R^2^ 0.0647, p < 0.001			R^2^ 0.0815, p < 0.001		
Mothers’ sedentary time weekend (n = 301)						
Fathers’ sedentary time weekend (mins)	0.37	0.25 to 0.49	<0.001	0.38	0.26 to 0.49	<0.001
N media items in household				−0.98	−3.15 to 1.18	0.365
	R^2^ 0.1335, p < 0.001			R^2^ 0.1419, p < 0.001		

^a^Adjusted for the total number of media items reported to be in the house, mother’s age and BMI, and the household IMD

The odds of a mother watching more than 2 h TV on a weekday were associated with an threefold increase if the father also watched this amount (OR 2.97; 95 % CI 1.79 to 4.91) compared with those where the father watched under 2 h TV per day (Table 4). At weekends the odds increased fivefold (OR 5.09; 95 % CI 3.30 to 7.86).

**Table 4:** Logistic regression of mothers’ TV viewing predicted by fathers’ TV viewing

	Unadjusted			Adjusted^a^		
	OR	95 % CI	p	OR	95 % CI	p
Mothers’ weekday TV (n = 325)						
Fathers’ weekend TV viewing	3.19	1.93 to 5.27	<0.001	2.97	1.79 to 4.91	<0.001
N media items in household				1.03	0.98 to 1.08	0.277
	Pseudo R^2^ 0.0509, p < 0.001			Pseudo R^2^ 0.0663, p < 0.001		
Mothers’ weekend TV (n = 325)						
Fathers’ weekend TV viewing	5.27	3.36 to 8.27	<0.001	5.09	3.30 to 7.86	<0.001
N media items in household				1.05	1.01 to 1.09	0.012
	Pseudo R^2^ 0.1109, p < 0.001			Pseudo R^2^ 0.1459, p < 0.001		

Reference category: <2 h per day

^a^Adjusted for the total number of media items reported to be in the house, mother’s age and BMI, and the household IMD

Weekday use of computers by mothers was positively associated with fathers’ use (OR 2.12; 95 % CI 1.30 to 3.41) (Table 5). On weekend days the direction of association between parents’ PC use was reversed; the odds of mothers using a PC for more than 30 min per weekend day was halved if the father used a PC for this amount of time (OR 0.45; 95 % CI 0.22 to 0.94) compared with those whose partners used a PC for 30 min or less per weekend day.

**Table 5:** Logistic regression of mothers’ PC use predicted by fathers’ PC use

	Unadjusted			Adjusted^a^		
	OR	95 % CI	p	OR	95 % CI	p
Mothers’ weekday PC (n = 324)						
Fathers’ weekday PC use	2.07	1.29 to 3.33	0.003	2.12	1.30 to 3.41	0.002
N media items in household				1.01	0.97 to 1.06	0.663
	Pseudo R^2^ 0.0221, p = 0.0025			Pseudo R^2^ 0.0267, p = 0.0559		
Mothers’ weekend PC (n = 325)						
Fathers’ weekend PC use	0.45	0.22 to 0.93	0.030	0.45	0.22 to 0.94	0.033
N media items in household				1.00	0.94 to 1.05	0.914
	Pseudo R^2^ 0.0178, p = 0.0298			Pseudo R^2^ 0.0320, p = 0.0459		

Reference category: ≤30 min per day

^a^Adjusted for the total number of media items reported to be in the house, mother’s age and BMI, and the household IMD

The odds for a mother using a smart phone were over five times higher on both weekdays (OR 5.96; 95 % CI 2.85 to 12.48) and weekend days (OR 5.28; 95 % CI 2.69 to 10.35) if the father also used a smart phone, compared with mothers where there was no use of a smart phone by the father (Table 6).

**Table 6:** Logistic regression of mothers’ smart phone use predicted by fathers’ smart phone use

	Unadjusted			Adjusted^a^		
	OR	95 % CI	p	OR	95 % CI	p
Mothers’ smart phone use weekday (n = 325)						
Fathers’ weekday smart phone use	6.48	3.25 to 12.90	<0.001	5.96	2.85 to 12.48	<0.001
N media items in household				1.06	1.01 to 1.11	0.014
	Pseudo R^2^ 0.1210, p < 0.001			Pseudo R^2^ 0.1400, p < 0.001		
Mothers’ smart phone use weekend (n = 325)						
Fathers’ weekend smart phone use	5.94	3.14 to 11.28	<0.001	5.28	2.69 to 10.35	<0.001
N media items in household				1.06	1.02 to 1.11	0.009
	Pseudo R^2^ 0.1067, p < 0.001			Pseudo R^2^ 0.1276, p < 0.001		

Reference category: no use

^a^Adjusted for the total number of media items reported to be in the house, mother’s age and BMI, and the household IMD

## Discussion

The results presented here show that for parents of young children, mothers’ sedentary time was strongly associated with that of fathers, particularly at weekends when parents are more likely to be at home together. Mothers’ TV viewing time on weekdays was also strongly positively associated with that of fathers, and this association was stronger at weekends. Smart phone use showed similarly strong associations on both types of day. The positive association for weekday PC use between dyads was reversed at weekends when the odds of a mother using a PC for more than 30 min was halved if the father used a PC for this amount of time. Adjusting the model for the number of PCs rather than the total number of media items did not change associations.

Parental sedentary time was positively associated on weekdays and weekend days with a stronger association at weekends. The lower amount of sedentary time at weekends compared with weekdays for both mothers and fathers might be explained by employment patterns and the sedentary nature of many jobs. On weekend days, mothers were over five times as likely to spend more than 2 h per day watching TV if the father did likewise (OR 5.27; 95 % CI 3.36 to 8.27). Since parents are likely to spend time together at weekends the magnitude of this association suggests that SV might be a behaviour that parents share when they are at home together, although this cannot be confirmed since times of viewing were not recorded. We have previously shown that child SV was associated with that of both mother and father [9]. The combined results provide evidence for the need to develop family based interventions or interventions aimed at both partners in order to reduce parental sedentary time and screen viewing behaviours.

The finding that mothers are 55 % less likely to use a PC for more than 30 min at the weekend if the father uses it for more than 30 min (compared with those where the father uses a PC for less than 30 min) is surprising. A plausible explanation, however, is that PCs tend to be shared within a household and used by only one person at a time. Alternatively, it might be that one parent is using a computer whilst the other parent is entertaining the children or is doing household chores. A third possibility is that, in contrast to TV viewing which tends to be a social behaviour, PC and laptop leisure activities, such as playing games, online shopping, or using social media sites, might not be reflected in the preferences of the partner.

Previous studies that have examined spousal associations in behaviours have mostly concentrated on PA at times of transitions in life. For example, a qualitative study found that whilst spouses had similar attitudes towards an active retirement, attitudes towards regular activity diverged, and shared participation in activity was rare [16]. However, spousal support was perceived to be important for participation in regular exercise. A randomized trial designed to increase physical activity and improve nutrition in recently co-habiting young couples provided some evidence that a short-term health promotion programme was associated with improvements in some health related behaviours [17]. In one of the few observational studies that has examined PA in young couples, marriage was found to have no impact on PA compared with remaining single [6]. After the birth of a child however, males’ physical activity decreased by 6 h per week compared with those who remained childless. In contrast, there was no effect of having a child on PA for females [6]. Our results complement the existing literature by showing that sedentary time and screen viewing are both associated in parents of school-aged children. It is unclear from our study to what extent this association is a result of assortative mating (choosing a partner with similar characteristics) versus being due to a shared household environment. A previous study found a link between longer duration of sharing a household and higher obesity and obesity-promoting behaviours, which suggests that a common environment in a partnership may play a more significant role than assortative mating [18]. Irrespective of the mechanism for such associations, we have provided evidence of a need to develop interventions that encourage at least one adult in the household to decrease sedentary time and become more active.

### Strengths and limitations

A strength of this study is the availability of sedentary time and SV behaviours for parent dyads along with the number of media devices in the home. This has allowed us to examine how the fathers’ behaviour is associated with that of the mother within the same household. One of the greatest strengths of the study is the objectively measured sedentary time. To our knowledge, this is the first study that has objectively measured PA in both parents of 5 and 6 year old children, the results of which have been published elsewhere [19]. The study is not, however, without its limitations which might reduce the impact of some of the conclusions. Screen viewing was self-reported by both parents. Such self-reported behaviour is likely to be under-estimated although the extent to which this is the case is unknown. However, any under-estimation should not affect the magnitude of the associations between SV of parents if it is assumed that the degree of under-reporting is relatively similar within dyads. Likewise, the number of media devices is self-reported and has not been objectively verified. A further limitation is concerned with the cross-sectional design of the study which does not allow the direction of association to be confirmed. Whether mothers influence fathers, or whether the reverse is true, is unclear. Our analysis has predicted mothers’ behaviour based on the behaviour of the child’s father; it would have been possible to assess associations in the opposite direction, but due to a lack of fathers’ information on the required co-variates, the resulting sample size would have been further reduced. A third alternative is that the association is bi-directional at different times and for different families, depending on life circumstances. Finally, as indicated in Table 1, the number of included participants is a relatively small subset of the whole sample. This is due to the limited number of families in which both parents were willing to participate in the study, and in particular where both parents were willing to wear accelerometers. Despite this, the results are a valuable contribution to the literature which is currently lacking in information about both parents of children.

## Conclusions

Sedentary time and SV behaviours are independently associated in parents of young children. Our results supplement the literature of the relationships in spousal behaviours and provide support for the development of interventions that promote a healthy lifestyle in families. In particular, the development of interventions that encourage at least one adult in the household to decrease sedentary time and become more active, particularly at weekends, could ultimately be of benefit to the entire family.
